# Home Treatment of Tuberculosis

**Published:** 1907-07

**Authors:** J. E. Summerfield

**Affiliations:** Atlanta, Ga.


### HOME TREATMENT OF TUBERCULOSIS.

By J. E. Summerfield, Atlanta, Ga.

In considering the various methods of treating tuberculosis, I think there is no doubt but that the greatest benefit is derived from such systematic treatment as can only be obtained in a properly conducted sanatarium. The wonderful results accomplished there can readily be understood when we consider how carefully each patient is watched. Under the constant observation of the physician, the least unfavorable symptom is noted and immediate attention given, and often a severe touble is thus averted. In the sanitarium given, and often a severe trouble is thus averted. In the sanitarium the patient receives minute instruciions how to care for himself. The amount of exercise to be taken is prescribed. He is shown the importance of proper ventilation, fresh air and sunshine, also the necessity of nourishing food, and finally he is taught how to prevent infection in others and re-infection in his own case. Then when he has completely recovered, or sufficiently improved so as to be able to leave the institution, he is prepared to take care of himself properly and to teach those with whom he comes in contact how to protect themselves. Unfortunately, however, but few can avail themselves of this method of treatment for two reasons; firstly, the comparatively few sanatoria and, secondly, the inability of the majority to pay the fees necessary to maintain such an institution. In many of the European countries the various cities are establishing sanatoria for the benefit of their citizens. In Germany especially, they are aided in this work by the sick benefit societies of the working people and also by the government. Statistics are kept, and it has been found the number cured and the number of others whose lives have been prolonged have saved the State millions of dollars. Even the larger life insurance companies are making use of the sanatoria for the benefit of their tuberculous policy holders. The question is being seriously agitated in this country, and now about ten states have their sanatoria, and we trust that in the not far distant future Georgia will also be able to assist her citizens in the same manner.

In the majority of cases, when the diagnosis of tuberculosis is made. the advice given by the doctor is that the patient seek a more favorable climate, either warmer or cooler as suits his condition. The advice is very easily given, but does the physician realize what this change means? In many instances, the breaking of family ties, traveling to a strange place with probably little or no ready money, no means to take care of himself and perhaps a family left at home needing his support. Is it a wonder if such patients become disheartened and homesick, grow worse and either become victims of the disease there or return home to die? Only a small number of patients can avail themselves of this form of treatment, and it should be advised only when all the conditions are favorable.

And now we come to the great majority who are neither able to seek treatment in a sanatorium nor able to go to a more suitable climate. And what can we do to aid them? During the past few years I have been giving special attention to this class of patients, and find that with patients in the incipient stage very good results can be obtained here, and with this advantage your patient does not become homesick and the expense incurred is very much less. The home patients must be divided into two groups, those in the incipient stage and those further advanced. As most of the home patients are compelled to support themselves, I have not found it only advising those whose occupations are too confining and harmful, to change them, and if possible, to do outside work. Most of these patients first consult the doctor in his office, and he rarely knows the condition of the patient's home. And it is here where all possibe attention should be given, for I have found that no matter how carefully you instruct your patients about home affairs but few will carry out your instructions. I make it a rule to visit the homes of such patients at intervals and see that the instructions are carried out. The amount of labor entailed is great, but the reward in the shape of a cured or arrested case is greater, and one feels amply repaid when he sees a father restored to health and again able to support his family, or a mother able to resume her duties

When such a patient is first seen and the diagnosis made, I think it best to be frank with him and tell him he has tuberculosis, that it is a curable disease and that the cure will depend more upon him than upon the attention of the doctor, and if he is not willing to obey instructions that treatment would be useless. As a rule there is little difficulty experienced in getting the co-operation of the patient. Examine the home carefully, see if the house is sufficiently high above the ground and dry, that the bed room is properly ventilated and the bed so placed that the patient does not sleep in a draft; otherwise place a screen around the bed to protect the patient. If possible have the patient sleep in a room alone. Knopf, of New York, has suggested an apparatus which is a modification of a tent, an arrangement whereby patients can sleep indoors but still breath outside air. The bed is placed alongside of a window which has the lower sash raised entirely. The apparatus is attached to the inside frame of the window in the same manner as an awning is fastened on the outside, and it can be raised or lowered by means of a pulley and rope. It is made of canavs and has a transparent window of celluloid enabling the patient to see what is gong on in the room and permitting those in the room to observe the patient. When lowered it covers the upper half of the patient, who protects his head and shoulderes by means of a woolen hood, leaving only the face exposed. In the daytime the apparatus can be pulled up and is out of the way. For patients confined to the bed, the apparatus can be used by day as well as by night. It has not been patented and can be made cheaply. It is described and cuts of same are shown in the Journal of the American Medical Association for Januay 19, 1907.

When patients are first seen a careful record of the conditions found is kept, and examinations are made weekly. I find that the short talk of encouragement given each patient at the examinations is of benefit. Microscopic examinations of the sputum are made and are only of value when positive. I have had cases with typical symptoms where repeated examinations of the sputum were negative.

EXERCISE:—The amount of exercise to be taken will depend upon the condition of the patient; in many cases deep breathing exercises are sufficient.

Fresh air and sunshine are also necessary, and the value of same must be impressed upon him.

EXPECTORATION:—Patients are forbidden to expectorate upon the floors. Have them place cuspidors containing an antiseptic solution in each room, and insist upon them making use of same.

CLOTHING:—It is necessary that the clothing of patients be of proper weight and warmth for the season. As a rule I find that most patients wear more clothing than necessary.

DIET:—Special attention must be given to the diet, and only easily digested and nourishing food should be used. My chief reliance is placed upon milk and raw eggs, usually beginning with a quart of milk and three to four raw eggs daily, soon increasing until the patient takes half a gallon or more of milk and eight to ten raw eggs daily. When patients state that milk disagrees with them and they feel bloated, I find that a small amount of citrate of soda dissolved in the milk will prevent this condition. The beneficial effects of milk and eggs can usually be observed by the second or third week.

MEDICATION:—As a rule medication is of minor importance and is prescribed to meet special symptoms, such as night sweats, hemoptysis, pain, cough, etc. In most cases I found it necessary to give a placebo, as patients receiving no medicine think they are not having proper attention given them. and I usually give these patients a capsule containing gnaiacol carbonate.

Will only report one case, as it is typical of most patients seen in the earlier stages.

Mrs. M., age 30, came under my care in May, 1905.

FAMILY HISTORY:—Both parents living. Two sisters, one brother and her first husband died of tuberculosis. Second husband living and well. About end of 1904 noticed that she was gradually losing weight, had a cough, night sweats, pain in chest.

EXAMINATION: — Anæmic — Weight 116 pounds. Diminished breathing on left side of chest. Percussion, dullness over apex of left lung, and on ausculation moist rales heard anteriorly and posteriorly over left apex. Temp. 100 deg. F. Visited her home, found it damp and advised her to move, which she did. Careful instructions were given her, as to fresh air, ventilation, cuspidors, etc., and nourishing food was prescribed. At first she took one quart of milk and four raw eggs daily and in three weeks was taking half a gallon of milk and ten raw eggs daily. She improved rapidly and by December, 1905, her weight was 142 pounds. Her general appearance was changed and many of her friends hardly recognized her. An examination recently made shows that the dullness has disappeared and only normal breathing is heard. I forgot to mention that the first examination of the sputum was positive, but several examinations made within the past six months were negative. The only medication given was a capsule containing gnaiscol carbonate with occasionally a light laxative added to counteract constipation.

803 Century Building.

